# Surgical Supervisor Feedback Affects Performance: A Blinded Randomized Study

**DOI:** 10.7759/cureus.1276

**Published:** 2017-05-25

**Authors:** Assad Zahid, Jonathan Hong, Christopher J Young

**Affiliations:** 1 Institute of Academic Surgery, Royal Prince Alfred Hospital, Sydney; 2 Colorectal Unit, Royal Prince Alfred Hospital

**Keywords:** feedback, surgery, task performance

## Abstract

While performing a simple task of following: a suture while closing a surgical wound in a simulated environment, we hypothesized that negative reinforcement results in increased procedural errors, longer operating time and poorer trainee satisfaction. We aimed to measure the effect on participant performance and the perception of the instructor, following positive or negative supervisor feedback during the task. A blinded randomized study was conducted assessing positive and negative supervisor feedback styles on participant performance in a simulated operation room. Students performed the task twice, with a reflection in between the repeated task. We found that the change in procedure time between the two tasks was adversely affected by feedback style. Participants receiving negative feedback sought cues to improve. From this study, it was found that negative supervisor feedback has the potential to adversely affect elements of performance. Despite this, participants receiving negative feedback express a willingness to improve their performance by seeking cues from the supervisor.

## Introduction

Stress in the operating room (OR) can adversely affect performance. Stressors can arise from many sources, including challenges in the operative procedure, interactions with supervisors and other staff and self-assessment of performance. With the current evolution of surgical training from the master-apprentice model to competency based training, there is also a culture change in the way trainees receive feedback [1]. There is a greater emphasis on reflection and structured feedback on performance utilizing validated tools implemented by training boards [2]. Previously, feedback was imparted by the supervisor either during or after a procedure with the quality and content of the feedback largely dependent on the supervisor. In some cases, the feedback may inspire the trainee to reflect and improve or may be negative and demoralizing. We aim to assess the impact of positive and negative feedback styles on the performance of participants in the operating room while assisting in performing a simple wound closure.

## Materials and methods

Ten medical students (six male and four female) in the first two years of training, with equivalent exposure to the operating room (OR) environment, were recruited and randomly allocated to receive positive or negative feedback from a surgeon while assisting the closure of a surgical wound. This study was performed in a fully functional simulated OR. All recruited students were informed that the study would involve their participation in closing a surgical wound.

An instructional video was produced for this study informing the students about how to best assist a surgeon while they are closing a surgical wound (Video 1).

**Video 1:** Instructional video informing medical students how to assist while closing a simulated surgical wound

This video was recorded in the same simulated OR in which the study occurred. The emphasis of the video was to ensure that the technique of assisting was transferred to the students. Information on optimal suture holding length at one-third of the distance from the wound was given. Furthermore, students were advised to avoid “loops, bombs, drags or snares.” Loops result from holding the suture too low, allowing the remaining suture length in front of the wound to loop and obstruct the surgeon's view and access to suturing the wound. A bomb was dropping the suture too early. A drag is not releasing the suture in time and causing a tug on the suture while the surgeon is attempting to pull the suture through. Finally, a snare is allowing a knot or the suture to be caught in a surgical instrument. All students watched the video once before the commencement of the study. The study commenced with students being randomly allocated to receive either positive or negative supervisor feedback.

Randomization was done utilizing a computer generated code. The students were not informed of the feedback style they would receive. Once randomized, students first assisted the surgeon in the closure of a 10 cm surgical wound. The surgeon was given a script of five sentences that he could use to provide feedback for each group (Appendix 1). This ensured that all feedback was standardized between the groups.

Once the wound was closed, the student then underwent a debriefing with a separate surgeon-supervisor where they were asked to reflect on the procedure. Gibbs’ cycle of structured debriefing was used as a guide to performing the reflection [3]. This learning from doing or experiential learning cycle aims to link the actions and thoughts involved in an experience with a six stage reflection tool. The stages of this structured debriefing include description, feelings, evaluation, analysis, conclusion and action plan [3]. The exact questions and statements made by the surgeon-supervisor performing the reflection task to the students can be found in Appendix 2. The supervisor performing the debriefing was also blinded to the feedback style the student received.

After the reflection, students performed the procedure once more and received the same feedback that they were allocated to. Finally, after the second procedure, the students were asked to complete a survey of their experience. Due to the small size of this study and to avoid confounding, the repeat negative-negative or positive-positive only feedback groups were chosen for allocation. In future studies, we will expand numbers and study alternate feedback patterns such as negative-positive and positive-negative pairings. All wound closure tasks were video recorded and analyzed to obtain data for the duration of the wound closure and the number of errors performed. The audio transcripts of the reflection were analyzed by two organizing members of the study to identify themes that were expressed by the students about their experience in the OR.

Grounded theory methodology was utilized to develop the main themes being expressed by the students [4]. Grounded theory is a methodological guideline developed within sociology, used mostly for the analytic assessment of qualitative data. It informs researchers of analytical categories, the identification of themes in their data, and conceptualizing the studied experience. The researcher codes categorize the qualitative data from the full interview transcription [4].

The primary endpoint was to assess whether feedback style impacted on the procedure duration. Secondary endpoints included the number of errors, themes from reflection task, participant feedback and supervisor feedback score of participants. Ethics for this study was approved by the hospital ethics review board and participants consented to involvement in the study.

Data were collected and entered using Microsoft Excel and analyzed using GraphPad Prism version 7 (La Jolla, San Diego, California, USA). The Mann-Whitney U-test was used to compare the two groups.

## Results

Timing

The average time for completion of the procedure was 159 seconds in the positive feedback group and 143 seconds in the negative feedback group. This difference was not significant (p=0.47). After the reflection tool, the mean procedure time was 120 seconds and 139 seconds in the positive and negative groups respectively (p=0.14).

**Figure 1:**
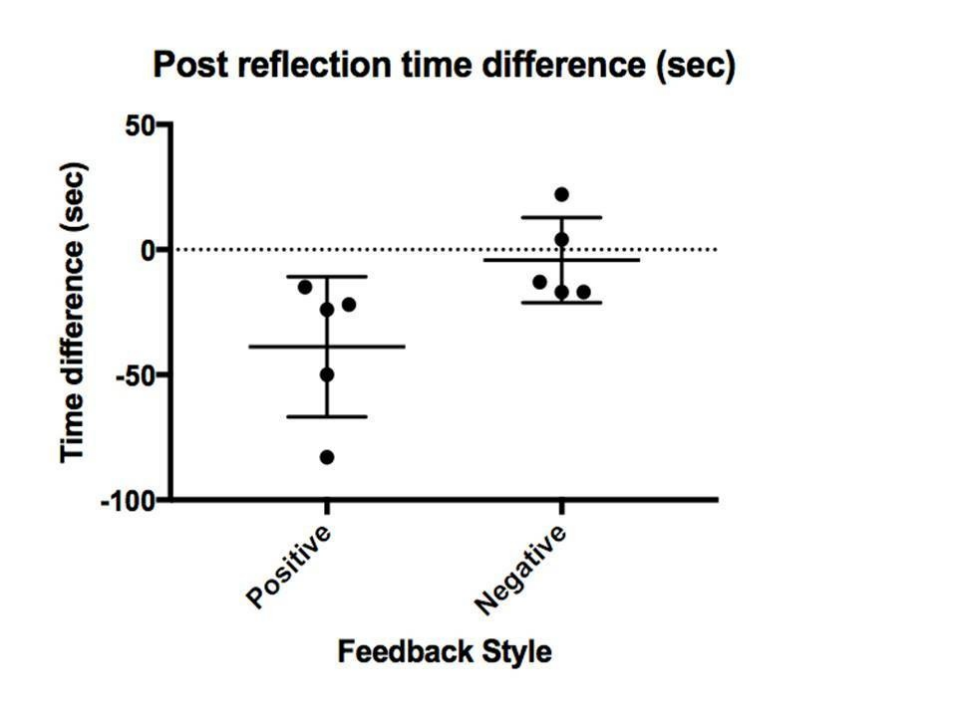
Difference in procedure time between groups after reflection tool

When comparing the change in time between the first and second procedures for the positive and negative feedback groups, the difference was significant (p= 0.03) (Figure 1). The positive feedback group improved on average 39 seconds between the two procedures, while the negative feedback group improved on average by four seconds. All participants in the positive feedback group were faster completing the second procedure, while two out of five participants in the negative feedback group took longer.

Errors

Errors were measured by reviewing recorded footage of each of the participants. Errors were scored for every “loop, bomb, drag or snare” that was performed. There was no statistical difference between the two groups (p=0.81)

Key themes during reflection tool

All reflection sessions were audio recorded, transcribed and subsequently reviewed by the study organizers. Key themes were noted and compared between the two analyses. This grounded theory approach lead to the development and understanding of key themes that the participants from each group expressed (Table 1). Participants receiving positive feedback felt supported and comfortable with the task. Negative feedback participants generally felt intimidated. Also, those receiving negative feedback sought feedback from the supervisor with an attempt to improve performance; this was not expressed by the positive feedback group.

**Table 1:** Key themes between positive and negative feedback groups

Positive Feedback	Negative Feedback
Relaxed	Intimidating
Uncertainty	Uncertainty
Supported	Nervous
Reassured	Seeking feedback

Supervisor feedback

The surgeon performing the task also provided a subjective score (out of five, with one being very poor and five reflecting an excellent performance) of the individual participants for every procedure. Initial score differences were not significant (p=0.59). After the debriefing, there was no statistical difference between the groups (p=0.17). However, it was noted that in the positive feedback group, four of the five participants improved their score by one point with one participant keeping the same score. In the negative feedback group, one participant lost one point, three maintained the same score and one improved by one point. One notable statement made by the surgeon during the task was the observation, that the participants in the negative group sought feedback on performance from the surgeon while this was not actioned by participants in the positive group.

Self-evaluation

When asked about individual performance, positive feedback participants stated their performance was average or above average. Negative feedback participants felt their performance was average or below average (p=0.12). When asked about how they would perform in future procedures, four out of five participants stated that they would perform better in the positive feedback group. While in the negative feedback group, one participant stated that he/she would perform extremely well, two stated better performances, and the other two stated the same performance.

## Discussion

Traditional surgical teaching has followed the master-apprentice model, where the trainee observes and performs procedures under the guidance of a supervisor who instructs them and provides feedback of their performance. This, however, is not standardized as surgeons’ are not trained as teachers, but are clinicians. Most trainees subsequently may not receive the same quality of feedback from their supervisors which subsequently will impact on their skills growth. Also, the style of feedback will have an impact on the mindset of the student, whether it promotes a growth mindset or not. With working hours being legislated and the subsequent decrease in exposure to the operating room, the quality of supervisor feedback is essential to trainee development [2,5].

We assume the absence of a significant difference in mean procedure time between the two groups that was most likely a reflection of the small sample size in the study. The slower procedure time for two negative feedback group students in the repeated task is likely based on the need of the participants to seek a means of improvement and feedback from the supervisor as mentioned by many in their post-study survey. We believe the use of the reflection tool provided insights into the way participants felt about the task [6]. Key themes were identified and an approach for the subsequent task was expressed. The key differences lay in reassuring and relaxed feeling felt by participants in the positive feedback group. The negative feedback group felt intimidated and nervous. Both groups felt uncertainty. This may be due to the participants not being given a clear objective to improve on for the study.

A major difference between the two groups was that the negative feedback group sought to improve by seeking feedback from the supervisor. This was not expressed by the positive feedback group, who were reassured of their performance and were happy to continue, even if they had made more errors than participants in the negative feedback group. Further research into supervisors with different personality types will be required, as with different participant personality types, but that is the subject of much larger studies than this one describes.

Eva & Regehr propose learners make better use of external information about their performance by self-directed assessment seeking processes where one takes personal responsibility for looking outward and explicitly seeking feedback from external sources [7]. This experiential development of skill is also reflected in Kolb’s learning theory. Kolb in 1984 described his experiential learning theory into four stages. Initially, a new experience is encountered. This is followed by reflective observation where inconsistencies between experience and understanding are resolved. This reflection gives rise to a new idea leading to the development of a new technique or modification of an existing process. Finally, there is the application of this step to their practice [8]. This utility of effective and not necessarily negative feedback seems essential to trigger such learning behavior.

No student in the positive feedback group improved more than one point in the surgeon’s assessment, potentially highlighting that there is a limitation to always giving positive feedback. Many of the positive feedback participants stated that they were feeling “babied” and “reassured” about their performance. This may ultimately mean they were less stressed and able to perform better rather than judging themselves like the negative feedback group. Hence there is a need for a balance in the feedback style to ensure performance enhancement with a growth mindset [9].

The current reform of surgical training is the greater emphasis on the work done by the Royal College of Physicians and Surgeons with the introduction of a competency-based training system, CanMEDS [10]. Rather than the traditional focus on the time-based training models, trainees progress once they have achieved competency in key themes. The implementation of this new system of training into a traditional supervisor mindset demands reforms to the way training and feedback are delivered to improve efficiency and effectiveness [11]. This study highlights that there is a role in the style of feedback delivery and the outcome of this on the trainee.

The limitations of this study is that it was conducted as a pilot study with only two groups of five participants, making it significantly underpowered. However, we strongly believe that the purpose of this study is qualitative and highlights the perceived impact of feedback style on trainee performance. The data from this study will be used to establish a larger sample sized study to validate the findings. Despite the small numbers, a significant result in the time of procedure change scores was noted. Supervisor scores were subjective and could represent a tougher scoring on the negative feedback group. To minimize biases, all participants were given very little information of the task before attending the session. This ensured that there was minimal difference between the two groups.

## Conclusions

This study has shown that negative supervisor feedback has the potential to adversely affect elements of performance. Despite this, participants receiving negative feedback express a willingness to improve their performance by seeking cues from their supervisor. This study emphasizes that we are still in search of the ‘Goldilocks” feedback strategy where performance is improved without the cloud of complacency.

## Appendices

Appendix 1.

Standardised Supervisor Comments

Hello. Can you please assist me with the closure of this wound?

I will be closing this wound with a continuous suture. The scissors are here (points to scissors).

Negative feedback candidates:

Commence with: Please avoid "loops, bombs, drags or snares" Please don't pull on the suture No loops please Don't drop the suture You need more practice

Positive feedback candidates:

Commence with: Thank you for helping me with this wound closure. Well done That is correct (if asked by the student if they are doing ok) Excellent Thank you for your help today, that was great.

Appendix 2:

Structured Debriefing after first round

Recording interview

Description:

What happened? Don’t make judgements yet, just describe the event/experience.

Feelings:

What were your reactions and feelings?

Evaluation:

What was good or bad about the experience? Make value judgements.

Analysis:

What sense can you make of the situation? What was really going on? Are there patterns or themes emerging? Bring ideas from other experiences to inform this analysis.

Conclusions (general):

What can be concluded, in a general sense from these experiences and analyses you have undertaken?

Conclusions (specific):

What can be concluded about your specific performance?

Personal action plans:

What are you going to do differently in this type of situation next time? What steps are you going to take on the basis of what you have learnt?
